# THE IMPACT OF SLEEP QUALITY ON NEUROCOGNITIVE FUNCTIONING IN CAREGIVERS OF PERSONS WITH DEMENTIA

**DOI:** 10.1093/geroni/igae098.2423

**Published:** 2024-12-31

**Authors:** Lauren Chrzanowski, Lauren Elliott, Sydnie Schneider, Keegan Diehl, Amir Abu-Samaha, Volker Neugebauer, Jonathan Singer

**Affiliations:** Texas Tech University, Lubbock, Texas, United States; Texas Tech University, Lubbock, Texas, United States; Texas Tech University, Lubbock, Texas, United States; Texas Tech University, Lubbock, Texas, United States; Texas Tech University, Lubbock, Texas, United States; Texas Tech University Health Sciences Center, Lubbock, Texas, United States; Texas Tech University, Lubbock, Texas, United States

## Abstract

Studies have found poor sleep quality is linked to decreased neurocognitive functioning in older adults. Notably, it has been found that older adults who exhibit worse sleep quality have decreased processing speed. However, research with caregivers is extremely limited and current research lacks the use of validated measures and neurocognitive assessments across several functional domains (e.g., processing speed; executive functioning). Further, most of these studies have been conducted with large urban samples. This study included family caregivers of persons with dementia in Lubbock County, which includes a small urban city and multiple rural cities. We compared Pittsburgh Sleep Quality Index (PSQI) scores with Trails Making Test delta (i.e., processing speed), Repeatable Battery for the Assessment of Neuropsychological Status (RBANS; i.e., overall neurocognitive functioning), and the Wisconsin Card Sorting Test (WCST; standardized percentiles of perseverative errors and conceptual level responses; i.e., executive functioning). There was no significant relationship between sleep and processing speed (r=-.173, p=.143), executive functioning (i.e., WCST standardized percent perseverative errors (r=-.219, p=.090); WCST standardized percent conceptual level responses (r=-.256, p=.059)), and overall neurocognitive functioning (r=-.110, p=.269). Interestingly, in this unique population of family caregivers there appears to not be a relationship between sleep and neurocognitive functioning in the domains examined. More research regarding sleep and cognition in family caregivers is needed, particularly that which addresses the interplay of sleep and other psychosocial variables impacting cognition in caregivers.

